# Supplementary material for: Fibrinogen Glycation and Presence of Glucose Impair Fibrin Polymerization—An In Vitro Study of Isolated Fibrinogen and Plasma from Patients with Diabetes Mellitus
Source: Biomolecules. 2020 Jun 7;10(6):877. doi: 10.3390/biom10060877 (PMC7356284; doi:10.3390/biom10060877)
Supplement: Supplementary file 1 [file biomolecules-10-00877-s001.pdf]

biomolecules

MDPI

Article

Fibrinogen glycation and presence of glucose impair fibrin polymerization – an *in vitro* study of isolated fibrinogen and plasma from patients with diabetes mellitus

Boguslawa Luzak[1]*, Magdalena Boncler[1], Marcin Kosmalski[2], Ewelina Mnich[1], Lidia Stanczyk[1], Tomasz Przygodzki[1] and Cezary Watala[1]

[1] Department of Hemostasis and Hemostatic Disorders, Chair of Biomedical Sciences, Medical University of Lodz, Poland, Mazowiecka 6/8, 92-216 Lodz, Poland
[2] Department of Clinical Pharmacology, 1st Chair of Internal Medicine, Medical University of Lodz, Poland
* Correspondence: boguslawa.luzak@umed.lodz.pl; Tel.: +48 42 2725740

1. Materials and Methods

Clot retraction

Clot retraction in the presence of 5 or 30 mM glucose was estimated according to Tucker et al. [1] with some modifications. Platelet rich plasma (200 µl of 3 x 10^8 platelets/ml in plasma) was mixed with mixed with red blood cells (2.5 µL) and Tyrode's buffer was added to make the final volume to 0.5 ml. Additionally, glucose was added to mixture to final concentrations of 5 mM or 30 mM. The control sample was prepared without glucose. Thrombin at final concentration of 1 U/mL (human thrombin from Enzyme Research Laboratories, United Kingdom) was added to initiate fibrin clot formation. Then, clot at 37°C was observed for 120 minutes and photographed (t0 – immediately after thrombin addition; t120 - 120 min after thrombin addition). Clot weight, as a marker of clot retraction, was measured at t120. Pictures were processed with Image-J software and clot surface areas were plotted. Data were expressed as a percentage of clot retraction (relative clot volume) = (area t0 − area t120)/(area t0) × 100.

2. Results

2.1. Analysis of human fibrinogen isolated from plasma of diabetic patients and control subjects

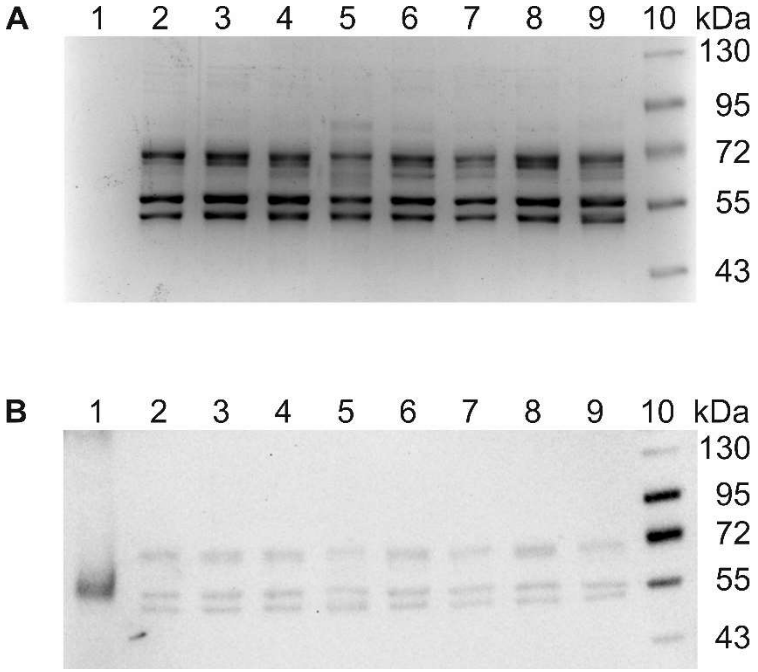

Figure S1. SDS-PAGE and Western blot analysis of human fibrinogen isolated from plasma of diabetic patients and control subjects. A) Representative SDS-PAGE gel showing the fibrinogen polypeptide chains (α, β, γ) stained with Coomassie brilliant blue. B) Representative Western blot image demonstrating CML-modified fibrinogen in the examined samples. Lanes: 1) positive control (CML-modified bovine serum albumin, 10 ng), 2-5) fibrinogen isolated from control subjects, 6-9) fibrinogen isolated from diabetic patients, 10) prestained protein ladder.

2.2. Determination of clot retraction in the presence of glucose

Table S1. Clot retraction in plasma samples during *in vitro* incubation with glucose

	Control (no glucose)	Glucose 5 mM	Glucose 30 mM
Clot retraction [%]	62.5 ± 10.8	68.1 ± 8.1, *P*=0.165	56.8 ± 7.6, *P*=0.071
Clot weight [mg]	14.3 (13.5; 17.5)	16.4 (12.5; 20.2), *P*=0.678	28.7 (20.0; 35.8), *P*=0.086

Data shown as mean ± SD for clot retraction or as median (interquartile range Q1; Q3) for clot weight (n=3). It was observed that the presence of glucose did not significantly modified clot retraction (data were analyzed with bootstrap-boosted procedures of t Student test for clot retraction, or with Wilcoxon test for clot weight).

3. References

1. Tucker, K.L.; Sage, T.; Gibbins, J.M. Clot retraction. *Methods Mol Biol* **2012**, *788*, 101-107, doi:10.1007/978-1-61779-307-3_8.

© 2020 by the authors. Licensee MDPI, Basel, Switzerland. This article is an open access article distributed under the terms and conditions of the Creative Commons Attribution (CC BY) license (http://creativecommons.org/licenses/by/4.0/).
